# Advances in in vitro folliculogenesis in domestic ruminants

**DOI:** 10.21451/1984-3143-AR2018-123

**Published:** 2020-05-22

**Authors:** José Ricardo de Figueiredo, Jesús Cadenas, Laritza Ferreira de Lima, Regiane Rodrigues Santos

**Affiliations:** 1 Laboratory of Manipulation of Oocytes and Preantral Follicles, Faculty of Veterinary, State University of Ceara, Fortaleza CE, Brazil.; 2 Schothorst Feed Research, Lelystad, The Netherlands.

**Keywords:** folliculogenesis, preantral follicle, ruminant, *in vitro* development

## Abstract

The *in vitro* follicle culture (IVFC) represents an outstanding tool to enhance our understanding of the control of folliculogenesis and to allow the future use of a large number of immature oocytes enclosed in preantral follicles (PFs) in assisted reproductive techniques in humans as well as in others mammalian species including the ruminants. So far, the best results of IVFC were reported from mice with the production of live offspring from primordial follicles cultured in vitro. Live birth has been obtained after the in vitro culture of bovine early antral follicles. However, in other ruminant species, these results have been limited to the production of a variable number of mature oocytes and low percentages of embryos after in vitro culture of goat, buffalo and sheep isolated secondary preantral follicles. The present review presents and discusses the main findings, limitations, and prospects of in vitro folliculogenesis in ruminants focusing on bovine, caprine, and ovine species.

## Introduction

Ruminants are distributed worldwide and have social and economic importance for many countries, contributing to circa 76% of the global livestock biomass (FAO, 2010). To keep animal production within expected levels, nutrition and reproduction are the key factors. Regarding reproduction, it is essential to understand the mechanisms involved in the formation of the gametes, as well as their development to improve techniques and outcomes (Thatcher, 2017).

At birth, ruminant ovaries contain thousands of immature oocytes, being the vast majority of them enclosed in preantral follicles, that represent the main ovarian oocyte reserve. Despite this large follicle population, most of them (approximately 99.9% of the follicles) will undergo atresia during folliculogenesis. The regulation of folliculogenesis in the preantral follicle phase is an extremely complex process and involves the interaction among endocrine, paracrine and autocrine factors, as previously revised (Figueiredo *et al*., 2011, 2018).

The Assisted Reproductive Techniques (ARTs) are of great importance for both basic and applied research. Basic, or fundamental, research is crucial to understand the physiology of reproduction (Smith *et al*., 2014). Applied research helps to overcome severe infertility either in male or female, as well as to increase the genetic selection rate of highly producing animals (Tan *et al*., 2017). Among the ARTs that aim to optimize the use in the future of the large ovarian oocyte reserve it is important to highlight the *in vitro* follicle culture (IVFC) (Green and Shikanov, 2016; Figueiredo *et al*., 2018). This technique represents an outstanding tool to enhance our understanding of the control of folliculogenesis and to allow the future use of a large number of immature oocytes enclosed in PFs in ARTs in humans as well as in others mammalian species. Interestingly, the production of live offspring from primordial follicles cultured *in vitro* has been successfully achieved in mice, and it was first reported in 1989 (Eppig and Schroeder, 1989). However, in ruminants, the results have been limited to the production a low percentage of embryos after *in vitro* culture of goat, buffalo, and sheep secondary preantral follicles (Silva *et al*., 2016). Therefore, this review presents and discusses the main findings, limitations, and prospects of *in vitro* folliculogenesis in ruminants focusing on bovine, caprine, and ovine species.

## Overview of follicle structure and populations and folliculogenesis regulation *in vivo*

It is well known that mammalian ovaries contain from thousands to millions of follicles whereby approximately ninety percent of this population is represented by preantral follicles usually classified as primordial, intermediate, primary and secondary follicles. Despite this large follicle population, the vast majority of them will be eliminated by a physiological process called atresia during folliculogenesis. Folliculogenesis is the physiological process of activation, growth, and maturation of the ovarian follicle. The regulation of folliculogenesis involves a complex interaction among endocrine, paracrine and autocrine factors which in turn affects steroidogenesis, angiogenesis, basement membrane turnover, follicular atresia, oocyte growth, and maturation as well as the proliferation and differentiation of follicular cells (Figueiredo *et al*., 2018). In the ovary, the distribution of the regulating factors (ligands and their corresponding receptors) varies among follicular compartments (oocyte, granulosa, and theca cells) and significant changes in gene expression pattern among follicular categories (Yoon *et al*., 2006) have been reported. The control of folliculogenesis is extremely complex because the regulating factors act by binding to different types of receptors that activate distinct signaling pathways and, sometimes different ligands share the same receptors (Figueiredo *et al*., 2018). Also, there are complex interactions among cell signaling pathways which eventually control gene expression that determines cell survival or death, quiescence or proliferation. Therefore, follicular maturation or atresia will depend on a delicate balance between stimulatory and inhibitory stimuli. Folliculogenesis during the preantral follicle phase can be divided into three steps: (i) the activation (recruitment) of primordial follicles, i.e., transition from primordial (quiescent follicle) to growing follicles (intermediate and primary follicle); (ii) development of primary and secondary follicles; (iii) transition from preantral to antral follicle. In general, it has been stated that the growth of primordial follicles up to the early antral stage is pituitary independent, being probably controlled by autocrine/paracrine mechanisms and modulated by gonadotrophins (for review see Figueiredo *et al*. 2018). With the understanding of the factors involved in the early folliculogenesis, it will be possible to optimize the use of the large oocyte ovarian reserve in ARTs in humans as well as in other mammalian species. Among these technologies, it is important to highlight the IVFC, as discussed in the coming sections.

## *In vitro* follicle culture (IVFC)

### Purpose, applications, and type of culture systems

Taking into account that the vast majority of follicles will be eliminated by atresia in case they remain in the ovary the ultimate goal of IVFC is to rescue preantral follicles from the ovary before they become atretic, and culture them up to maturational stages for further *in vitro* fertilization and embryo production, functioning as an artificial ovary. This technology has some current and future applications such as: (i) to study the control of early folliculogenesis; (ii) to complement other reproductive technologies (e.g., *in vitro* embryo production, nuclear transfer, etc); (iii) to create gamete banks from endangered species and breeds; (iv) to preserve the fertility in individuals subjected to cancer treatment; infertility treatment (human), and (v) to aid in studies on reproductive toxicology (Figueiredo *et al*., 2011).

Basically, there are two ways to culture preantral follicles: in the isolated form or enclosed in ovarian tissue. Isolated follicles can be cultured in a two-dimensional system which means the follicle is placed on the surface, such as plastic or on an extracellular matrix for instance collagen gel or follicles can be cultured in a 3D system enclosed in an extracellular matrix such as alginate. In situ cultures, on the other hand, have been performed using ovarian fragments or the whole ovary (Figueiredo *et al*., 2011).

### Main endpoints used to evaluate the efficiency of IVFC

**Figure 1:**
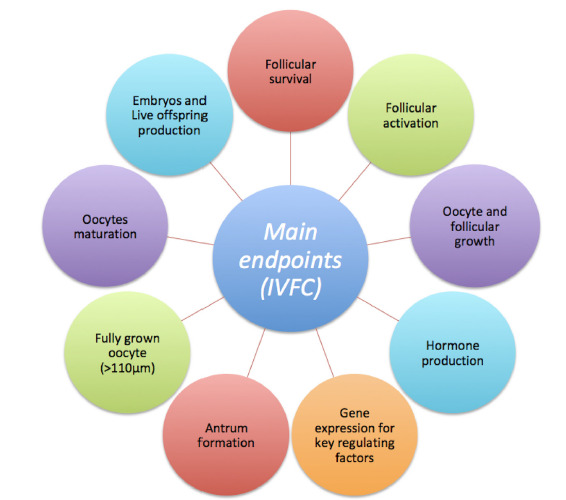
The main endpoints used to evaluate the efficiency of IVFC in mammals.

The efficiency of IVFC can be evaluated using the following endpoints that are crucial for understanding folliculogenesis regulation: follicular survival (morphology/viability); follicular activation and progression through folliculogenesis; oocyte and follicular growth; hormone production; gene expression for key factors (Ligands/receptors); antrum formation; production of fully grown (> 110µm) meiotically competent oocytes; finally, it is also possible to evaluate the oocyte developmental competence through the production of embryos and live offspring (Fig. 1).

## Main progresses in the *in vitro* culture of ruminant follicles

### Bovine

Although live birth has been produced after the *in vitro* culture of cumulus-granulosa cell complexes from early antral follicles (0.3 - 0.7 mm in diameter) (Hirao *et al*., 2004; Yamamoto *et al*., 1999), the bovine species is the one facing more difficulties when it comes to move forward in the field of IVFC. The best achievement so far from PFs is the antrum formation after the *in vitro* culture of primordial and intermediate follicles (< 40 µm diameter) enclosed in ovarian tissue into secondary follicles (~110 µm in diameter), followed by the isolation and *in vitro* culture of those *in vitro* grown secondary follicles (McLaughlin and Telfer, 2010). Araújo *et al*. (2014b) made a profound review on this subject, however, significant advances have been published in recent years that are worth mentioning. The main results are summarized in Table 1.

Early PFs, i.e., primordial and primary follicles are usually cultured *in vitro* enclosed in ovarian tissue (in situ). With this system, Jorssen *et al*. (2014) showed that neutral red (NR) staining can be used to facilitate follicle evaluation during a short-term culture (6 days) without affecting follicle developmental competence regardless the oxygen tension (high: 20% O2; low: 5% O2). Regarding culture media supplements, the addition of either growth and differentiation factor 9 (GDF-9) or basic fibroblast growth factor (bFGF) to a medium containing follicle stimulating hormone (FSH) enhanced the beneficial effect of FSH alone in terms of follicle morphology, activation and growth (follicle diameter from ~ 25 µm on D0 to ~ 90 µm on D22 of culture) (Tang *et al*., 2012).

In addition, the base medium itself can affect follicle viability and development. In fact, Jimenez *et al*. (2016) stated that αMEM is more effective maintaining follicle viability and promoting follicle growth than both TCM199 and McCoy media. Conversely, Castro *et al*. (2014) recommended the use of TCM199 and McCoy media for the culture of fresh and vitrified bovine ovarian tissue, respectively, meaning that the process to which the ovarian tissue is submitted prior culture must also be considered before selecting a base culture medium. 

Besides growth factors and hormones, the role of cytokines during folliculogenesis, such as tumor necrosis factor-alpha (TNF-α) and interleukin-1 (IL-1), as well as their distribution within the bovine ovary has been investigated. Thereby, proteins of the TNF-α system members, i.e., TNF-α and its receptors (TNFR1/TNFR2), have been detected in oocytes from all follicular categories, in granulosa cells from the secondary stage onwards, and in theca cells at the antral stage. Nonetheless, the addition of TNF-α has shown to reduce follicle survival after 6 days of culture (Silva *et al*., 2017a). Moreover, proteins of the IL-1 system, i.e., IL-1β and its receptors (IL-1RI, IL-1RII, and IL-1RA) have been detected in oocytes and granulosa cells from all follicular categories and in theca cells at the antral stage. But unlike TNF-α, the addition of IL-1β favored follicular activation and development after 6 days of culture (Passos *et al*., 2016). The in situ system has also complemented other biotechnologies such as ovarian tissue xenotransplantation. As a matter of fact, a 24 h culture of bovine ovarian tissue in the presence of VEGF prior to xenotransplantation into mice enhanced follicle survival for up to 2 weeks (Langbeen *et al*., 2016). 

As seen in the in situ system, bovine follicles cultured *in vitro* in the isolated form are also affected by the base medium composition. Rossetto *et al*. (2012) obtained greater follicle growth, viability and antrum formation (60%), when using TCM199 medium compared to both α-MEM and McCoy media with the same supplementations and medium replacement regime i.e., half of the medium (75 µl) was refreshed every 4 days. However, when another medium replacement regime was used (addition of 5 µl of fresh culture medium to an initial volume of 50 µl every other day), α-MEM became equivalent to TCM199 in terms of follicular growth and antrum formation (Araújo *et al*., 2015).

Furthermore, different supplements have shown to exert a positive effect on follicular growth and/or antrum formation of isolated secondary follicles when added alone, such as: FSH (Passos *et al*., 2013; Silva *et al*., 2014), bone morphogenetic protein-15 (BMP-15) (Passos *et al*., 2013), VEGF (Araújo *et al*., 2014a), insulin (Rossetto *et al*., 2016), and alpha lipoic acid (ALA) (Zoheir *et al*., 2017). Nevertheless, the combination of FSH and BMP-15 or Activin A have not improved the IVFC outcome (Passos *et al*., 2013; Silva *et al*., 2014, respectively). 

Less developed follicular categories, i.e., primordial and primary follicles have successfully reached the antral stage *in vitro* after 21 days of culture: primordial follicles (< 40 µm) cultured in a two-step system consisting on 6 days of in situ culture followed by 15 days of isolated 2D culture (Act A was added to isolated follicles) (McLaughlin and Telfer, 2010); and primary follicles (50-70 µm) in an isolated 3D system (collagen matrix) in the presence of FSH, luteinizing hormone (LH), estradiol (E2), epidermal growth factor (EGF) and bFGF (Sun and Li, 2013).

In summary, bovine PFs have been able to remain viable during *in vitro* culture and to form antrum from the primordial stage. Nevertheless, no oocyte meiotic maturation has been accomplished yet. Therefore, future research should focus on the specific factors involved on the *in vitro* obtention of metaphase II oocytes in this species. Furthermore, besides the fact that bovine is indicated as model for reproductive toxicology studies, especially during oocyte maturation (Santos *et al*., 2014), not so much has been done with PFs.

### Ovine

Among the three main species of domestic ruminants (bovine, ovine and caprine), the ovine species is the one where the IVFC has improved the most in the last years (Table 2), probably because is the most used animal model for humans. Hence, it has been reported the production of a relatively high rate of metaphase II (MII) oocytes (Arunakumari *et al*., 2010; Barboni *et al*., 2011) and a low number of embryos at the morula stage after *in vitro* fertilization (IVF) or parthenogenetic activation of *in vitro* cultured isolated secondary follicles (Arunakumari *et al*., 2010; Barboni *et al*., 2011; Luz *et al*., 2013). In this sense, Arunakumari *et al*. (2010) obtained 68% of MII oocytes from which 25% developed to the 2-cell embryo stage, and 16% reached the morula stage (out of the cleaved embryos) after the IVF of oocytes derived from the isolated PFs (200-400 µm) cultured in medium TCM 199 containing thyroxin (T4), FSH, IGF-I, and GH for 6 days. Barboni *et al*. (2011) also produced competent oocytes from smaller PFs (170 µm) cultured for 12 days in α-MEM with fetal calf serum (FCS) and FSH, although after IVF only 10% of the resulting embryos reached >16-cell stage. Interestingly, *in vitro* grown oocytes from 360 µm early antral follicles (AFs) (final follicle diameter at the end of the culture) presented similar methylation pattern and developmental capability than their *in vivo* grown counterparts (early AFs with the same diameter). However, oocytes from both *in vivo* and *in vitro* grown early AFs (360 µm) presented low competence compared to oocytes from *in vivo* grown AFs (6 mm) (Barboni *et al*., 2011).

Aiming to optimize the current IVFC systems and to understand better the process of folliculogenesis *in vitro,* several substances and/or culture systems have been tested mainly on isolated secondary follicles (> 200 µm). Thus, it has been shown that rutin alone could potentially replace the combination of the three commonly used antioxidants in culture medium (transferrin, selenium and ascorbic acid), and consequently simplify its composition (Lins *et al*., 2017). Also, leukemia inhibitory factor (LIF) promoted the rupture of the basement membrane without affecting oocyte maturation or embryo development since 8-cell parthenotes were produced (Luz *et al*., 2012). The association of LIF and kit ligand (KL), on the other hand, stimulated oocyte meiotic resumption and even a morula was produced after IVF. Nevertheless, this association did not improve the results obtained by LIF alone in terms of oocyte maturation and embryo production (Luz *et al*., 2013). Likewise, the addition of human leptin to the culture medium increased follicular daily growth but did not affect oocyte maturation (Kamalamma *et al*., 2016).

Despite all these advances on IVFC of secondary follicles, oocyte maturation and embryo production rates are still far below the results obtained from follicles entirely grown *in vivo*. On this regard, several studies with this follicular category have shown that IVFC (isolated follicles in a 2D system) negatively affect the expression pattern and/or level of genes related to oocyte survival and development such as P450 aromatase (Lakshminarayana *et al*., 2014); B-cell leukemia/lymphoma -2 (Bcl2) and Bcl2-associated X protein (Bax) (Praveen Chakravarthi *et al*., 2015); connexins 32 and 43 (CX32 and CX43) (Chakravarthi *et al*., 2016a); cyclin B1 (CCNB1) and cyclin D1 (CND1) (Chakravarthi *et al*., 2016b); and GDF-9 and BMP-15 (Kona *et al*., 2016). 

Early PFs (primordial and primary follicles) are usually cultured in situ (Bertoldo *et al*., 2014), although they have been cultured in the isolated form as well within a 3D matrix (alginate) (Sadeghnia *et al*., 2016). These follicular categories have provided some knowledge about what factors mediate follicle activation. Nowadays, increasing evidence suggests that some of them pertain to transforming growth factor β (TGF-β) superfamily (Knight and Glister, 2006), which includes the bone morphogenetic proteins (BMPs). Nonetheless, the addition of BMP4 during the culture of ovarian cortex pieces did not affect follicle activation but enhanced follicle survival and growth (Bertoldo *et al*., 2014). Also, the stiffness of the environment surrounding the follicles seems to affect their activation and further development. The encapsulation of ovarian cortex in 0.5 or 1% alginate was detrimental for follicle development, while the encapsulation of isolated primordial follicles in 2% alginate potentiated their growth (Sadeghnia *et al*., 2016).

As it was introduced above, one of the multiple potential applications of IVFC is to serve as an *in vitro* model for toxicology assays. It is noteworthy that in this species, this technique has been used already for this purpose. Thus, it was determined the minimum concentration of some metabolic stressors that impaired preantral follicle function: 300 µM ammonia, 8 mM urea, 210 µM non-esterified fatty acids (NEFA) (30 µM stearic acid + 60 µM palmitic acid + 120 µM oleic acid), and 0.75 µM β-hydroxybutyric acid (BHB) (Nandi *et al*., 2017). Moreover, it was shown that aqueous extracts of the plant Justicia insularis were able to maintain follicle morphology and to stimulate primordial follicle activation during the *in vitro* culture of PFs enclosed in ovarian tissue for 7 days (Mbemya *et al*., 2017). Early PFs (primordial and primary follicles) are usually cultured in situ (Bertoldo *et al*., 2014), although they have been cultured in the isolated form as well within a 3D matrix (alginate) (Sadeghnia *et al*., 2016). These follicular categories have provided some knowledge about what factors mediate follicle activation. Nowadays, increasing evidence suggests that some of them pertain to transforming growth factor β (TGF-β) superfamily (Knight and Glister, 2006), which include the bone morphogenetic proteins (BMPs). Nonetheless, the addition of BMP4 during the culture of ovarian cortex pieces did not affect follicle activation but enhanced follicle survival and growth (Bertoldo *et al*., 2014). Also, the stiffness of the environment surrounding the follicles seems to affect their activation and further development. The encapsulation of ovarian cortex in 0.5 or 1% alginate was detrimental for follicle development, while the encapsulation of isolated primordial follicles in 2% alginate potentiated their growth (Sadeghnia *et al*., 2016).

In general, in spite of the progress made on this topic for the last few years, the developmental competence of the oocytes obtained from *in vitro* cultured PFs is still low relative to their *in vivo* counterparts. Even though a considerable proportion of oocytes from PFs are nowadays able to grow and reach the MII stage *in vitro*, the fact that embryos are capable of developing only until morulae highlights the need for further investigation regarding oocyte cytoplasmic maturation. 

**Table 1:** Chronological advances in in vitro culture of bovine preantral follicles*.

References/Base medium	Follicle Category/Culture system	Tested compounds/Main findings
McLaughlin and Telfer, 2010 - McCoy´s 5a Bicarbonate (20mM Hepes, 3mM GLUT, 0.1% BSA, 2.5 µg/ml TRAN, 4 ng/ml SEL, 10 ng/ml INS and 50 µg/ml AA)	Primordial - *In situ* (6 days) Secondary - Isolated 2D (15 days)	100 ng/ml Act A or 50 ng/ml FSH - Primordial follicle - ↑ activation and growth; Act A - ↑ follicle and oocyte growth and antrum formation
Tang *et al*., 2012 - α-MEM (100 ng/ml FSH, 1.25 mg/ml BSA, 1 µg/ml INS, 5.5 µg/ml TRAN and 5 ng/ml SEL)	Preantral - *In situ* (22 days)	200 ng/ml GDF-9 or 100 ng/ml bFGF - GDF-9+FSH and bFGF+FSH - ↓ apoptosis and ↑ activation; GDF9+FSH - ↑ follicle diameter
Rossetto *et al*., 2012 - Base media (20mM Hepes, 3mM GLUT, 0.1% BSA, 2.5 µg/ml TRAN, 4 ng/ml SEL, 10 ng/ml INS, 50 µg/ml AA, 100 ng/ml Act A and 100 ng/ml FSH)	Secondary - Isolated 2D (16 days)	Different base media: αMEM, McCoy and TCM199 - TCM199 - ↑ follicle viability, diameter and antrum formation
Passos *et al*., 2013 - α-MEM (10 µg/ml INS, 5.5 µg/ml TRAN, 5 ng/ml SEL, 3 mg/ml BSA, 2mM GLUT, 2mM HYP, 50 µg/ml AA)	Secondary - Isolated 2D (12 days)	50 ng/ml BMP-15 and 50 ng/ml FSH - BMP-15 and FSH alone - ↑follicular volume and antrum formation; BMP-15+FSH - ↑ atresia
Sun and Li, 2013 - α-MEM (0.23 mM PYR, 1.5 mM GLUT, 2 mM HYP, 7.5% FBS, 5 µg/ml INS, 5 µg/ml TRAN, 5 ng/ml SEL)	Primary - Isolated 3D (Type I collagen - 21 days)	0.25 µg/ml FSH, 5 IU/ml LH, 0.5 µg/ml E2, 25 ng/ml EGF and 50 ng/ml bFGF - All factors - ↑ follicle diameter and antrum formation
Silva *et al*., 2014 - α-MEM (3 mg/mL BSA, 10 µg/ml INS, 5.5 µg/ml TRAN, 5 ng/ml SEL, 2 mM GLUT, 2 mM HYP, 50 µg/ml AA)	Secondary - Isolated 2D (18 days)	100 ng/ml Act A and FSH (50 ng/ml _D0-D6_; 100 ng/ml _D7-D12_; 200 ng/ml _D13-D18_) - Sequential FSH - ↑ follicle growth; Act A and FSH alone ↑ follicle survival.
Araújo *et al*., 2014a - α-MEM (3 mg/mL BSA, 10 µg/ml INS, 5.5 µg/ml TRAN, 6.7 ng/ml SEL, 2 mM GLUT, 2 mM HYP, 50 µg/ml AA, 100 ng/ml FSH)	Secondary - Isolated 2D/3D (alginate - 32 days)	100 ng/ml VEGF, 50 ng/ml GH, 50 ng/ml IGF-I - System 2D X 3D - VEGF + 2D: ↑ diameter, antrum formation and growth rate, and maintained morphology
Jorssen *et al*., 2014 - McCoy´s 5a (10mM Hepes, 3mM GLUT; 0.1% BSA, 5 µg/ml TRAN, 5 ng/ml SEL, 5 µg /ml INS, 50 µg/ml AA)	Preantral - *In situ* (6 days)	50 µg/ml neutral red (NR) in the presence of: 5% O_2_ vs. 20% O_2_ - Follicle dynamics are not influenced by O_2_ tension
Castro *et al*., 2014 - Base media (3mM GLUT; 2mM HYP, 0.1% BSA, 2.5 µg/ml TRAN, 4 ng/ml SEL, 10 ng/ml INS, 50 µg/ml AA)	Preantral - *In situ* (5 days)	Different base media: McCoy, α-MEM, and TCM199 (Fresh vs. Vitrified) - TCM199 maintained morphology (Fresh tissue); McCoy ↑ growth and maintained viability (Vitrified tissue)
Araújo *et al*., 2015 - α-MEM or TCM199 (3 mg/mL BSA, 10 µg/ml INS, 5.5 µg/ml TRAN, 5 ng/ml SEL, 2 mM GLUT, 2 mM HYP, 50 µg/ml AA)	Secondary - Isolated 2D (32 days)	α-MEM vs. TCM199 (60µl-Replacement - C vs. 5 µl-addition - S) - ↑diameter and antrum formation in TCM199-C vs α-MEM-C.
Rossetto *et al*., 2016 - TCM-199-HEPES (3mM GLUT, 0.1% BSA, 2.5 µg/ml TRAN, 4 ng/ml SEL, 10 ng/ml INS, 50 µg/ml AA, 100 ng/ml Act A, 100 ng/ml FSH	Secondary - Isolated 2D and 3D (Collagen - 18 days)	5, 10 ng/ml or µg/ml INS (2D) and 10 ng/ml INS+ FSH fixed (100 ng/ml) vs. sequential (1 ng/ml_D0-D6_; 10 ng/ml_D6-D12_; 100 ng/ml_D12-D18_) -10 ng/ml INS alone or with fixed FSH ↑ diameter and daily growth.
Jimenez *et al*., 2016 - Base media (20 mM Hepes, 3mM GLUT, 0.1% BSA, 2.5 µg/ml TRAN, 4 ng/ml SEL, 10 ng/ml INS, 50 µg/ml AA	Preantral - *In situ* (7 days)	Different base media: αMEM, TCM199 or McCoy - αMEM showed better results regarding follicle viability and growth
Passos *et al*., 2016 - α-MEM (2 mM GLUTA, 2 mM HYPO, 3 mg/ml BSA, 10 µg/ml INS, 5.5 µg/ml TRANS, 5 ng/ml SEL)	Preantral- *In situ* (6 days)	1, 10, 50 and 100 ng/ml IL-1β - 10 ng/ml IL-1β - ↑developing follicles and maintained normal morphology
Silva *et al*., 2017a - α-MEM( 2 mM GLUTA, 2 mM HYPO, 1.25 mg/ml BSA, 10 µg/ml INS, 5.5 µg/ml TRANS, 5 ng/ml SEL)	All follicular categories - *In situ* (6 days)	1, 10, 100 or 200 ng/ml TNF-α and 1, 10, 100 or 200 ng/ml dexamethasone - TNF-α ↑ cell apoptosis and 10 ng/ml dexamethasone conserved follicle ultrastructure
Zoheir *et al*., 2017 - TCM-199 (10% Newborn Calf Serum, 0.23 mM PYRU, 1% ITS, 2.2 g/l sodium bicarbonate, 100 ng/ml FSH, 100 ng/ml EGF	Secondary - Isolated 2D (15 days)	100, 250 or 500 µM ALA - ALA ↑follicle growth and maintained viability

*All the results are compared to control group. Abbreviations: 2D, two-dimensional culture system; 3D, three-dimensional culture system; BSA, bovine serum albumin; GLUT, glutamine; INS, Insulin; TRAN, Transferrin; SEL, Selenium AA, acid ascorbic; Hypo, Hypoxanthine; PYRU, Pyruvate; FBS, fetal bovine serum; ITS, commercial insulin, transferrin, selenium; FSH, follicle-stimulating hormone; Act A, activin A; GDF-9, growth and differentiation factor 9; bFGF, basic fibroblast growth factor; BMP-15, bone morphogenetic protein 15; LH, luteinizing hormone; EGF, epidermal growth factor; VEGF, vascular endothelial growth factor; IGF-I, insulin-like growth factor; GH, growth hormone; IL-1β, interleukin-1 β; TNF-α, tumor necrosis factor-alpha; ALA, alpha lipoic acid.

**Table 2:** Chronological advances in in vitro culture of ovine preantral follicles*.

References/Base medium	Follicle Category/Culture system	Tested compounds/Main findings
**Arunakumari *et al*., 2010** **-** TCM199 Bicarbonate	Secondary - Isolated 2D vs. 3D (Agar-coated plates - 6 days)	Different concentrations and association of ITS, IGF-I, insulin, GH, and TGF-β with 1 µg/ml T4 + 2 µg/ml FSH (Microdrops vs. Agar gel) - Combination of 1µg/ml T4, 2 µg/ml FSH, 10 ng/ml IGF-I, and 1 mIU/ml GH ↑ antrum formation, MII oocytes No differences between culture systems. 25% 2-cell embryo, and 16% morula.
**Barboni *et al*., 2011** - α-MEM (2% FCS, 1% ITS ,1 µg/ml FSH)	Secondary - Isolated 2D (14 days)	**Oocyte nuclear epigenetic maturation from early antral follicles grown (*in vivo* vs. *in vitro)* -** Similar methylation profile in *in vivo* and *in vitro.* Similar oocyte maturation, fertilization, and embryo production from *in vivo* and *in vitro* grown EAFs. 32% Fertilization, 90% <16-cell embryos and 10% >16-cell embryos
**Luz *et al*., 2012** - α-MEM(10 µg/ml INS, 5.5 µg/ml TRAN, 5.5 ng/ml SEL, 3 mg/ml BSA, 2 mM GLUT, 2 mM HYPO, 50 µg/ml AA)	Secondary - Isolated 2D (18 days)	10 and 50 ng/ml LIF alone or in combination with FSH (100 ng/ml _D0-D6_; 1000 ng/ml _D6-D18_; 200 ng/ml _D13-D18_) - 50 ng/ml LIF ↑extrusion. 1 Morula after IVF
**Luz *et al*., 2013** - α-MEM(10 µg/ml INS, 5.5 µg/ml TRAN, 5.5 ng/ml SEL, 3 mg/ml BSA, 2 mM GLUT, 2 mM HYPO, 50 µg/ml AA and 50 ng/ml LIF)	Secondary - Isolated 2D (18 days)	50 ng/ml IGF-I, and 50 ng/ml KL, alone or in combination - KL ↑oocyte meiotic resumption. 58.3% eight-cell stage parthenotes.
**Bertoldo *et al*., 2014** **-** Waymouth MB 752/1 (25 mg/l PYR, 6.25 µg/ml INS, 6.25 µg/ml TRAN, 6.25 µg/ml SEL, 1.25 mg/ml BSA, 5.35 µg/ml LA)	Primordial and primary follicles - *In situ* (6 days)	25, 50 or 100 ng/ml BMP4 with or without 50 ng/ml FSH -50 ng/ml BMP4 - ↑ follicle and oocyte diameters. Protected primordial follicles from apoptosis
**Lakshminarayana *et al*., 2014** **-** TCM199 (1µg/ml T4, 2.5 µg/ml FSH, 10 ng/ml IGF-I and 1 mIU/ml GH)	Secondary - Isolated 2D (6 days)	**Expression of P450 aromatase gene in cumulus cells and oocytes at different follicular stages (*in vivo* vs. *in vitro). In vitro*** culture ↓ P450 aromatase expression.
**Chakravarthi *et al*., 2015** - TCM199 (1µg/ml T4, 2.5 µg/ml FSH, 10 ng/ml IGF-I and 1 mIU/ml GH)	Secondary - Isolated 2D (6 days)	**Quantitative expression of *Bcl2* and *Bax* genes in cumulus cells and oocytes at different follicular stages (*in vivo* vs. *in vitro) - Bcl2*** to *Bax* ratio in oocytes and cumulus cells differed between *in vivo* and *in vitro* grown antral follicles
**Chakravarthi *et al*., 2016a** - TCM199 (1µg/ml T4, 2.5 µg/ml FSH, 10 ng/ml IGF-I and 1 mIU/ml GH)	Secondary - Isolated 2D (6 days)	**Quantitative expression of *CX32 and CX43* genes in cumulus cells and oocytes at different follicular stages (*in vivo* vs. *in vitro) - In vitro*** follicle culture ↓ expression of CX32 and CX43.
**Chakravarthi *et al*., 2016b** **-** TCM199 **(**1µg/ml T4**,** 2.5 µg/ml FSH, 10 ng/ml IGF-I, 1 mIU/ml GH)	Secondary - Isolated 2D (6 days)	**Quantitative expression of *CCNB1 and CCND1* genes in cumulus cells and oocytes at different follicular stages (*in vivo* vs. *in vitro) - In vitro*** follicle culture unbalanced the expression pattern of CCNB1 and CCND1
**Sadeghnia *et al*., 2016** **-** α-MEM (10 µg/ml INS, 5.5 µg/ml TRAN, 5.5 ng/ml SEL, 3 mg/ml BSA, 100 ng/ml FSH, 100 ng/ml GDF-9, 50 µg/ml AA)	Primordial and primary - *In situ* (8 days) and Isolated 3D (8 days)	0.5, 1 or 2% alginate (ovarian tissue and isolated follicles) - Alginate encapsulation ↓ number of secondary follicles. Encapsulation ↑follicle growth
**Kamalamma *et al*., 2016** - TCM199 (alone or supplemented by 1 µg/ml T4, 2.5 µg/ml FSH, 10 ng/ml IGF-I, 1 mIU/ml GH)	Secondary - Isolated 2D (6 days)	0-1000 ng/ml leptin and 10 ng/ml human leptin vs. 10 ng/ml ovine leptin - ↑growing follicles, diameter, antrum formation and oocyte maturationcin TCM199 with supplementation and 10 ng/ml human or ovine leptin.
**Kona *et al*., 2016** - TCM199 (1µg/ml T4, 2.5 µg/ml FSH, 10 ng/ml IGF-I, 1 mIU/ml GH)	Secondary - Isolated 2D (6 days)	**Quantitative expression of *GDF9 and BMP15* genes in cumulus cells and oocytes at different follicular stages (*in vivo* vs. *in vitro) -In vitro*** follicle culture altered the stage-specific changes in the expression of GDF9 and BMP15
**Lins *et al*., 2107** - α-MEM(10 ng/ml INS, 3 mg/ml BSA, 2 mM GLUT, 2 mM HYPO)	Secondary - Isolated 2D (12days)	0.1, 1 or 10 µg/ml rutin alone or in combination with 5.5 µg/ml TRA, 5 ng/ml SEL, and 50 ng/ml AA - Similar normal follicles (%) and fully-grown oocytes (%) between 0.1 µg/ml rutin alone and the combination of the three antioxidants
**Nandi *et al*., 2017** - α-MEM (1% ITS, 3 mg/ml BSA, 2 mM GLUT, 2 mM HYPO, 7 µg/ml FSH and 0.23 mM PYR)	Secondary - Isolated 2D (7 and 14 days)	Ammonia, urea, NEFA, and BHB - Minimum concentrations to impair follicle function.
**Mbemya *et al*., 2017** - α-MEM (10 µg/ml INS, 5.5 µg/ml TRAN, 5 ng/ml SEL, 1.25 mg/ml BSA, 2 mM GLUT, 2 mM HYPO)	Primordial and primary - *In situ* (7 days)	**50 ng/ml FSH vs. 0.3, 1.25, or 5 mg/ml *J.insularis*. 300 µg/ml Anethole with 0.3 mg/ml *J.insularis*, or 50 ng/ml FSH -** 0.3 ng/ml *J.insularis* maintained follicle morphology**.**

*All the results are compared to control group. Abbreviations: 2D, two-dimensional culture system; 3D, three-dimensional culture system; EAFs, early antral follicles; BSA, bovine serum albumin; GLUT, glutamine; INS, Insulin; TRAN, Transferrin; SEL, Selenium AA, acid ascorbic; Hypo, Hypoxanthine; PYR, Pyruvate; Bcl2, B-cell leukemia/lymphoma-2; Bax, Bcl2-associated X protein; CCNB1, cyclin B1; CCND1, cyclin D1; CX32, connexin 32; CX43, connexin 43; TGF-β, transforming growth factor-β; GDF-9, growth and differentiation factor 9; BMP-15, bone morphogenetic protein 15; BMP4, bone morphogenetic protein 4; FCS, fetal calf serum; ITS, commercial insulin transferrin selenium; FSH, follicle-stimulating hormone; IGF-I, insulin-like growth factor; GH, growth hormone; TNF-α, tumor necrosis factor-alpha; KL, Kit ligand; LIF, leukemia inhibitory factor; LA, linoleic acid; LPF, large preantral follicles; NEFA, non-esterified fatty acids; BHB, β-hydroxybutyric acid.

### Caprine

In comparison with the other two species reviewed in the present article, the caprine has been by far the most studied species regarding folliculogenesis *in vitro* in the last years (Table 3). A few 2- to 16-cell embryos have been produced from secondary follicles cultured in vitro in the isolated form (Saraiva *et al*., 2010; Silva *et al*., 2014), and even one has reached the morula stage after IVF (Magalhães *et al*., 2011). Despite that, oocyte maturation and embryo production rates are still low when compared to oocytes grown in vivo. In this regard, wishing to understand and improve conditions for successful IVFC in goats, different substances and/or culture systems have been tested on different follicular categories.

Isolated secondary follicles (~200 µm) improved their growth rates in the presence of increasing concentrations of FSH (100 ng/ml D0-D6; 500 ng/ml D6-D12; 1000 ng/ml D12-D18) alone (Saraiva *et al*., 2011) or in combination with LH and/or EGF (Saraiva *et al*., 2010; Silva *et al*., 2013). Moreover, sequential increasing FSH concentrations stimulated oocyte meiotic resumption to the GVBD stage when added alone (Saraiva *et al*., 2011) or in combination with IGF-I (Magalhães-Padilha *et al*., 2012b), while a low number of oocytes were able to reach the MII stage when sequential FSH was combined with: insulin (Chaves *et al*., 2012), IGF-II (Duarte *et al*., 2013), EGF (Silva *et al*., 2013), LH and EGF (Saraiva *et al*., 2010), GH (Magalhães *et al*., 2011), and VEGF (Araújo *et al*., 2011; Silva *et al*., 2014). Moreover, a few of these MII oocytes were fertilized and continued further development into 8-cell embryo (Silva *et al*., 2014), 16-cell embryo (Saraiva *et al*., 2010), and morula (Magalhães *et al*., 2011), which is the latest embryo developmental stage attained so far. 

Insulin and FSH are present in almost every culture medium for IVFC, although their concentration when combined is still focus of discussion (Dipaz-Berrocal *et al*., 2017; Paes *et al*., 2018). It has been suggested that 10 ng/ml insulin, a lower concentration than that which comes in the ITS composition, could be more efficient in promoting meiotic resumption in the presence sequential FSH (Chaves *et al*., 2012). However, some authors described that in the presence of either GH or VEGF, a high insulin concentration (10 µg/ml) combined with fixed 100 ng/ml FSH instead of sequential FSH can improve oocyte developmental competence (Ferreira *et al*., 2016; Silva *et al*., 2017b); and also stimulate antrum formation in the presence of phytohemagglutinin (PHA) (Cunha *et al*., 2013). Conversely, Ferreira *et al*. (2018) showed no positive effect of the association of high insulin and fixed FSH. This fact might be due to the source of FSH since Ferreira *et al*. (2018) used recombinant human FSH while most studies used recombinant bovine FSH. Conversely, fixed 10 mIU/ml human FSH improved oocyte meiotic resumption when compared to sequential bovine FSH (Rocha *et al*., 2014). Even the base medium itself can influence the follicular response to FSH and insulin. Hence, Amburana cearensis (Amb) ethanolic extract (0.2 mg/ml) base medium promoted higher follicle daily growth rate in the presence of sequential FSH and low insulin concentration than α-minimum essential medium (α-MEM) (Gouveia *et al*., 2016).

It is most likely that the effect of any supplement may also depend on follicular category since it has been shown that PFs and early antral follicles (EAFs) behave differently under the same culture conditions (Cadenas *et al*., 2017). Thus, human FSH increased follicle and oocyte diameters of EAFs (~350 µm) but did not affect PFs (Ferreira *et al*., 2018). Likewise, unlike PFs, EAFs have shown the greatest MII rate described so far from *in vitro* grown oocytes (46.2% calculated out of the total number of cultured follicles) in response to GH added to a medium with low insulin and no FSH (Cadenas *et al*., 2018). Also, Cadenas *et al*. (2018) were able to identify some non-invasive signs for the efficiency of IVFC for EAFs: follicle daily growth ≥ 6.1µm, follicle diameter ≥ 600.1µm, and oocyte diameter ≥ 120.1µm.

Besides culture media composition, many other factors have shown to affect the development of isolated early stage follicles such as the reproductive age of the ovary donor (prepubertal vs. adult), culture period, base media, and culture system (2D vs. 3D). Hence, PFs from prepubertal goats have reached the antral stage, but contrary to PFs from adult goats, were not able to produce MII oocytes after 18 days of IVFC (Amin *et al*., 2013; Silva *et al*., 2014) regardless the culture system, i.e., 2D vs 0.5% alginate (3D) (Silva *et al*., 2014). However, Brito *et al*. (2014) related a positive effect of a lower matrix stiffness (0.25% alginate) on follicle growth and oocyte meiotic resumption when compared to 0.5% alginate and 2D system. Furthermore, the coculture of 5 PFs per alginate bead stimulated follicle growth, and also a new matrix composed by 12.5 mg/ml fibrinogen and 0.125% alginate (fibrin-alginate) improved oocyte meiotic resumption when compared to 0.25% alginate (Brito *et al*., 2016).

The suitable culture period for IVFC in the isolated form is still a matter of debate. Even though 18 days is the most commonly used for large secondary follicles (Ferreira *et al*., 2016; Silva *et al*., 2017b), it seems that this follicular category may benefit from an extended culture period (30 to 36 days) (Pessoa *et al*., 2014), while 18 days has been described as the most suitable culture period for EAFs (Cadenas *et al*., 2018). 

Primordial and primary follicles have been usually cultured in situ. Within this culture system, several authors have reported the stimulation of follicle activation, and follicle and oocyte growth after a long-term culture (16 days) in the presence of: FSH during the first half (D0-D8) followed by either GH (Magalhães-Padilha *et al*., 2012a) or fibroblast growth factor-10 (FGF-10) during the second half (D8-D16) of the culture period (Almeida *et al*., 2015); KL during the first half and FSH during the second half of the culture (Lima *et al*., 2012); and also, FSH and IGF-I throughout the entire culture period, which turned up in increasing the percentage of secondary follicles (28%) (Magalhaes-Padilha *et al*., 2012). Other substances added for a shorter culture time (6 to 7 days) have also shown to exert a positive effect on follicle activation, survival and growth such as: the interaction between melatonin and FSH (Rocha *et al*., 2013); EGF (Lopes *et al*., 2015); KL (Faustino *et al*., 2013); and Concavalin A (Con A) (Portela *et al*., 2014). On the other hand, Keratinocyte growth factor-1 (KGF-1), also known as FGF-7, did not have a positive impact on early folliculogenesis *in vitro* (Faustino *et al*., 2013).

**Table 3:** Chronological advances in in vitro culture of caprine preantral follicles*.

References/Base medium	Follicle Category/Culture system	Tested compounds/Main findings
**Saraiva *et al*., 2010** - α-MEM (3 mg/ml BSA, 10 µg/ml INS, 5.5 µg/ml TRAN, 5 ng/ml SEL, 2 mM GLUT, 2 mM HYPO, 50 µg/ml AA, Sequential FSH)	Secondary - Isolated 2D (18 days)	50 or 100 ng/ml LH and 50, or 100 ng/ml EGF alone or associated - LH+EGF ↑ follicle growth and oocyte meiotic resumption. 2 embryos (8- and 16-cell) in 100 ng/ml LH+EGF
**Saraiva *et al*., 2011** **-** α-MEM (1.25 mg/ml BSA, 10 µg/ml INS, 5.5 µg/ml TRAN, 5 ng/ml SEL, 2 mM GLUT, 2 mM HYPO, 50 µg/ml AA)	Secondary - Isolated 2D (18 days)	Fixed (100, or 1000 ng/ml), Sequential FSH - Sequential FSH ↓extrusion, ↑ antrum formation and oocyte meiotic resumption.
**Magalhães *et al*., 2011** **-** α-MEM (3 mg/ml BSA, 10 µg/ml INS, 5.5 µg/ml TRAN, 5 ng/ml SEL, 2 mM GLUT, 2 mM HYPO, 50 µg/ml AA, Sequential FSH)	Secondary - Isolated 2D (18 days)	10 or 50 ng/ml GH - 50 ng/ml GH ↑ antrum formation and oocyte meiotic resumption. 1 morula from 50 ng/ml GH
**Araújo *et al*., 2011** **-** α-MEM (3 mg/ml BSA, 10 µg/ml INS, 5.5 µg/ml TRAN, 5 ng/ml SEL, 2 mM GLUT, 2 mM HYPO, 50 µg/ml AA, Sequential FSH)	Secondary - Isolated 2D (18 days)	10 or 100 ng/ml VEGF - 100 ng/ml VEGF ↑ oocyte meiotic resumption
**Chaves *et al*., 2012** **-** α-MEM (3 mg/ml BSA, 5.5 µg/ml TRAN, 5 ng/ml SEL, 2 mM GLUT, 2 mM HYPO, 50 µg/ml AA)	Secondary - Isolated 2D (18 days)	5 or 10 ng/ml, or 10 µg/ml insulin alone or in association to sequential - 10 ng/ml insulin with sequential FSH ↑ oocyte meiotic resumption
**Lima *et al*., 2012** **-** α-MEM (1.25 mg/ml BSA, 10 µg/ml INS, 5.5 µg/ml TRAN, 5 ng/ml SEL, 2 mM GLUT, 2 mM HYPO, 50 µg/ml AA)	Primordial and primary - *In situ* (16 days)	50 ng/ml KL and 50 ng/ml FSH alone or in different combinations - KL (D0-D8)/FSH(D8-D16) ↑activation and growth
**Magalhães-Padilha *et al*., 2012** - α-MEM (1.25 mg/ml BSA, 10 µg/ml INS, 5.5 µg/ml TRAN, 5 ng/ml SEL, 2 mM GLUT, 2 mM HYPO, 50 µg/ml AA)	Primordial and primary - *In situ* (16 days)	50 ng/ml IGF-I and 50 ng/ml FSH alone, or in different combinations - IGF-I+FSH ↑activation and growth, number of secondary follicles
**Magalhães-Padilha *et al*., 2012a** - α-MEM (1.25 mg/ml BSA, 10 µg/ml INS, 5.5 µg/ml TRAN, 5 ng/ml SEL, 2 mM GLUT, 2 mM HYPO, 50 µg/ml AA)	Primordial and primary - *In situ* (16 days)	10 ng/ml GH and 50 ng/ml FSH alone, or in different combinations - FSH (D0-D8)/GH (D8-D16) ↑ follicle morphology, viability, activation, growth and secondary follicles
**Magalhães-Padilha *et al*., 2012b** - α-MEM (3 mg/ml BSA, 10 µg/ml INS, 5.5 µg/ml TRAN, 5 ng/ml SEL, 2 mM GLUT, 2 mM HYPO, 50 µg/ml AA, Sequential FSH)	Secondary - Isolated 2D (18 days)	50 or 100 ng/ml IGF-I - IGF-I ↑ oocyte meiotic resumption. 50 ng/ml IGF-I ↑ antrum formation
**Cunha *et al*., 2013** - α-MEM (3 mg/ml BSA, 10 µg/ml INS, 5.5 µg/ml TRAN, 5 ng/ml SEL, 2 mM GLUT, 2 mM HYPO, 50 µg/ml AA, 100 ng/ml FSH)	Secondary - Isolated 2D (18 days)	1, 10, 50, 100 or 200 µg/ml PHA-10 µg/ml PHA maintained follicle ultrastructure and ↑antrum formation
**Duarte *et al*., 2013** - α-MEM (3 mg/ml BSA, 10 ng/ml INS, 5.5 µg/ml TRAN, 5 ng/ml SEL, 2 mM GLUT, 2 mM HYPO, 50 µg/ml AA)	Secondary - Isolated 2D (18 days)	20 or 50 ng/ml IGF-II, or sequential FSH - 20 ng/ml IGF-II alone or in associated to sequential FSH ↑ oocyte meiotic resumption ^†^
**Faustino *et al*., 2013** - α-MEM (1.25 mg/ml BSA, 10 µg/ml INS, 5.5 µg/ml TRAN, 5 ng/ml SEL, 2 mM GLUT, 2 mM HYPO, 50 µg/ml AA)	Primordial and primary - *In situ* (7 days)	1 ng/ml KGF-1 and 50 ng/ml KL alone or in combination - 50 ng/ml KL alone ↑ activation and growth
**Rocha *et al*., 2013** **-** α-MEM (1.25 mg/ml BSA, 10 µg/ml INS, 5.5 µg/ml TRAN, 5 ng/ml SEL, 2 mM GLUT, 2 mM HYPO, 0.23mM PYR)	Primordial and primary - *In situ* (7 days)	100, 250, 500, or 1000 pM melatonin, and 50 ng/ml FSH, alone or in combination - 1000 pM melatonin+50 ng/ml FSH ↑ follicular and oocyte diameters
**Silva *et al*., 2013** - α-MEM (3 mg/ml BSA, 10 µg/ml INS, 5.5 µg/ml TRAN, 5 ng/ml SEL, 2 mM GLUT, 2 mM HYPO, 50 µg/ml AA, Sequential FSH)	Secondary - Isolated 2D (18 days)	50 or 100 ng/ml EGF - EGF ↑ follicle daily growth rate. 50 ng/ml EGF ↑ oocyte meiotic resumption
**Amin *et al*., 2013** **-** Bicarbonate-buffered TCM199	Secondary - Isolated 2D (6 days)	Different concentrations and association of T4, FSH, GH, EGF, and IGF-I - All substances alone or in combination ↑ follicle growth and antrum formation. The association T4+FSH+GH+EGF ↑ extrusion.
References/Base medium	Follicle Category/Culture system	Tested compounds/Main findings
**Pessoa *et al*., 2014** - α-MEM (3 mg/ml BSA, 10 ng/ml INS, 5.5 µg/ml TRAN, 5 ng/ml SEL, 2 mM GLUT, 2 mM HYPO, 50 µg/ml AA, Sequential FSH and 50 ng/ml GH)	Secondary - Isolated 2D (Different days) and 3D (alginate - 36 days)	Different culture periods: 18, 24, 30, 36, or 42 days (2D vs. 3D) - Follicle diameter and oocyte meiotic resumption ↑ until day 36. 3D system did not affect oocyte meiotic resumption.
**Portela *et al*., 2014** **-** α-MEM (1.25 mg/ml BSA, 10 µg/ml INS, 5.5 µg/ml TRAN, 5 ng/ml SEL, 2 mM GLUT, 2 mM HYPO, 0.23mM PYR)	Primordial and primary - *In situ (*6 days)	5, 10, 20 or 10 µg/ml Con A. 10 µg/ml Con A and 50 ng/ml FSH, alone or in combination - 10 µg/ml Con A and 50 ng/ml FSH alone ↑activation.
**Silva *et al*., 2014** - α-MEM (3 mg/ml BSA, 10 µg/ml INS, 5.5 µg/ml TRAN, 5 ng/ml SEL, 2 mM GLUT, 2 mM HYPO, 50 µg/ml AA, Sequential FSH, 100 ng/ml VEGF, 1 mg/ml FET )	Secondary - Isolated 2D and Isolated 3D (0.5% alginate - 18 days)	Follicles from Adult Vs. Prepubertal ovaries (2D vs. 3D) - 3D system ↑ survival and ↓ oocyte extrusion. Greater follicle and oocyte and meiotic resumption diameter in 2D from adult ovaries. Four 2-cell embryos in 2D and one 8 cell-embryo in 3D.
**Rocha *et al*., 2014** - α-MEM (3 mg/ml BSA, 10 µg/ml INS, 5.5 µg/ml TRAN, 5 ng/ml SEL, 2 mM GLUT, 2 mM HYPO, 50 µg/ml AA, 100 ng/ml VEGF, 1 mg/ml FET)	Secondary - Isolated 3D (0.5% alginate - 18 days)	10 mIU/mlvhuman FSH Vs. Sequential FSH - 10 mIU/ml human FSH ↑oocyte meiotic resumption.
**Brito *et al*., 2014** **-** α-MEM (3 mg/ml BSA, 10 µg/ml INS, 5.5 µg/ml TRAN, 5 ng/ml SEL, 2 mM GLUT, 2 mM HYPO, 50 µg/ml AA, Sequential FSH )	Secondary - Isolated 3D (alginate - 18 days)	0.25%, 0.5%, or 1% alginate - 0.5% alginate maintained better follicle integrity. 0.25% alginate ↑ follicle diameter, daily growth and oocyte meiotic resumption.
**Almeida *et al*., 2015** **-** α-MEM (1.25 mg/ml BSA, 10 µg/ml INS, 5.5 µg/ml TRAN, 5 ng/ml SEL, 2 mM GLUT, 2 mM HYPO, 50 AA)	Primordial and primary - *In situ* (16 days)	50 ng/ml FGF-10 and 50 ng/ml FSH alone, in combination or sequentially - FSH(D0-D8)/FGF-10 (D8-D16) showed ↑ percentages of normal and growing follicles
**Lopes *et al*., 2015** **-** α-MEM (3 mg/ml BSA, 10 µg/ml INS, 5.5 µg/ml TRAN, 5 ng/ml SEL, 2 mM GLUT, 2 mM HYPO, 50 µg/ml AA)	Primordial and primary - *In situ* (6 days)	10 µg/ml PHA and 100 µg/ml EGF alone or in combination (Healthy goats Vs. CAEV) - EGF alone ↑ growth in both healthy and CAEV infected goats
**Gouveia *et al*., 2016** **-** α-MEM or different concentrations of Amb extracts	Secondary - Isolated 2D (12 days)	α-MEM Vs. Amb extracts (0.1, 0.2 or 0.4 mg/ml). α-MEM, 0.2 mg/ml Amb (3 mg/ml BSA, 10 µg/ml INS, 5.5 µg/ml TRAN, 5 ng/ml SEL, 2 mM GLUT, 2 mM HYPO, 50 µg/ml AA) or sequential FSH - 100ng/ml_D0-D6_, 500 ng/ml_D6-D12_,) - α-MEM and 0.2 mg/ml Amb similar morphology, antrum formation and follicle diameter
**Ferreira *et al*., 2016** **-** α-MEM (3 mg/ml BSA, 5.5 µg/ml TRAN, 5 ng/ml SEL, 2 mM GLUT, 2 mM HYPO, 50 µg/ml AA and 50 ng/ml GH)	Secondary - Isolated 2D (18 days)	10 ng/ml or 10 µg/ml insulin alone or associated to either fixed 100 ng/ml FSH or sequential FSH - 10 ng/ml insulin ↓extrusion. 10 µg/ml insulin ↑ follicle growth. 100 ng/ml FSH ↑oocyte meiotic resumption. 10 µg/ml insulin +100 ng/ml FSH ↑ mean oocyte diameter
**Brito *et al*., 2016** **-** α-MEM (3 mg/ml BSA, 10 µg/ml INS, 5.5 µg/ml TRAN, 5 ng/ml SEL, 2 mM GLUT, 2 mM HYPO, 50 µg/ml AA, Sequential FSH)	Secondary - Isolated 3D (alginate - 18 days)	Number of follicles per 0.25% alginate bead and beads per well. 5 follicles/bead in alginate, fibrin-alginate, or hyaluronate) - 5 follicles/bead ↑diameter. Alginate ↑follicle daily growth. Fibrin-alginate ↑ oocyte meiotic resumption.
**Silva *et al*., 2017b** **-** α-MEM (3 mg/ml BSA, 5.5 µg/ml TRAN, 5 ng/ml SEL, 2 mM GLUT, 2 mM HYPO, 50 µg/ml AA and 100 ng/ml VEGF)	Secondary - Isolated 2D (18 days)	10 ng/ml or 10 µg/ml insulin alone or associated to either fixed 100 ng/ml FSH or sequential - 10 µg/ml insulin ↑ follicle growth. 10 µg/ml insulin + fixed FSH ↑ oocyte meiotic resumption.
**Cadenas *et al*., 2017** **-** α-MEM (3 mg/ml BSA, 10 ng/ml INS, 5.5 µg/ml TRAN, 5 ng/ml SEL, 2 mM GLUT, 2 mM HYPO and 50 µg/ml AA)	Secondary and tertiary - Isolated 2D (24 days - PFs and 18 days EAFs)	50 ng/ml GH or 100 ng/ml VEGF alone, in combination or sequentially (Secondary PF vs. Tertiary EAF) - 50 ng/ml GH ↑ oocyte growth and meiotic resumption only in EAFs.
**Ferreira *et al*., 2018** **-** α-MEM (3 mg/ml BSA, 10 µg/ml INS, 5.5 µg/ml TRAN, 5 ng/ml SEL, 2 mM GLUT, 2 mM HYPO and 50 µg/ml AA)	Secondary - Isolated 2D (18 days)	10, 50 or 100 mIU/ml human FSH (PFs vs. EAFS) - No positive effect of human FSH on PFs. On EAFs:50 mIU/ml human FSH ↑ follicle and oocyte growth

*All the results are compared to control group. Abbreviations: 2D, two-dimensional culture system; 3D, three-dimensional culture system; PFs, preantral follicles; EAFs, early antral follicles; BSA, bovine serum albumin; GLUT, glutamine; INS, Insulin; TRAN, Transferrin; SEL, Selenium AA, acid ascorbic; Hypo, Hypoxanthine; PYRU, Pyruvate; FET, fetuin; LH, luteinizing hormone; EGF, epidermal growth factor; FSH, follicle-stimulating hormone; Sequential FSH - 100 ng/ml in D0-D6, 500 ng/ml in D6-D12,1000 ng/ml in D12-D18; GH, growth hormone; VEGF, vascular endothelial growth factor; KL, Kit ligand; IGF-I, insulin-like growth factor I; IGF-II, insulin-like growth factor II; PHA, phytohemagglutinin; KGF-1, keratinocyte growth factor 1; T4, thyroxine; Con A, concavalin A; FGF-10, fibroblastic growth factor 10; CAEV, caprine arthritis encephalitis lentivirus; Amb, Amburana cearensis extract.

Despite the undeniable progress obtained in goats, oocyte maturation and embryo production rates are still very low compared to oocytes originating from follicles grown *in vivo*, which must serve for encouraging further research on this topic. Overall, the data generated seem to point out the need to develop future dynamic and customized culture media for IVFC as the differences among follicles regarding their growth rates, and between follicular categories have shown to affect oocyte maturation *in vitro.*

## Future of IVFC: possible new strategies to overcome the current limitations

To further advance in efficient IVFC methods, some limitations and lack of information need to be overcome. For instance, the *in vitro* production of a fertilizable oocyte from a primordial follicle requires a long-term culture, which may affect oocyte quality and, consequently embryo prodution. Few studies are reported in animal species other than ruminants, and should be consider when improving culture techniques. It was indicated before that IVC of ovine preantral follicles yield oocytes with normal nuclear-epigenetic maturation (Barboni *et al*., 2011). However, this study was performed with secondary follicles and not starting from primordial ones, where it was shown in mice that deficiencies at transcriptional and epigenetic levels can occur (Wang *et al*., 2017). Importantly, IVFC of murine follicles is much shorter than that for large mammals, which can increase the urgence of studies at methylation level. It was demonstrated that apoptosis in murine primary oocytes is mediated by retrotransposon activity (Malki *et al*., 2014), and the suppresion of this activity is determined by DNA epigenetic modification (Findlay *et al*., 2015). In other words, methods to measure epigenetic risks, as well as to avoid them during IVFC are still needed. Besides this, preantral follicles are commonly cryopreserved for further *in vitro* culture. It is well known that the stress caused during exposure to cryoprotectants and the cooling process itself affect important organelles like the endoplasmic reticulum (ER) requiring the possible culture medium enrichment with antioxidants (Brito *et al*., 2013). Studying the ultrastructure of caprine preantral follicles, it was observed that atretic preantral follicles usually presented damaged ER (Silva *et al*., 2000).

## Final consideration

The control of the survival, activation and development of ruminant follicles *in vitro* is hugely complex and involves multiple interactions among extra and intraovarian factors and can be influenced by the type of base culture media, medium replacement regime, type of culture system (2D vs 3D), culture duration, ovarian source (pre-pubertal vs adult), extracellular matrix components, follicular categories (preantral vs early follicles). Unfortunately, these factors do not act on an isolated form, but interact with each other, making the development of IVFC protocols a challenge. Encouraging results have been reported including satisfactory rates of follicle survival, activation, antral formation and the production of fully grown meiotically competent oocytes especially in caprine and ovine species. However, the *in vitro* embryo production from *in vitro* grown oocytes is still low. Therefore, improvements in *in vitro* follicle culture system should be done to improve oocyte quality (oocyte developmental competence) for further production of viable offspring. This fact will allow the future use of a large number of immature oocytes enclosed in PFs in assisted reproductive technologies in humans as well as in others mammalian species.
